# Penile squamous cell carcinoma in neurofibromatosis type 1 (NF1): RAS-pathway mechanisms and management — A case report

**DOI:** 10.1016/j.eucr.2025.103257

**Published:** 2025-10-29

**Authors:** Babak Javanmard, Sina Samenezhad, Dorna Rafighi, Lena yaghoubpour, nasim Dehghan

**Affiliations:** a Urology Department, School of Medicine, Shahid Beheshti University of Medical Sciences, Tehran, Iran; b Department of Microbiology, Faculty of Basic Sciences, Research Sciences Branch, Islamic Azad University, Tehran, Iran; c Department of Pathology, Shohada-e-tajrish Educational Hospital, School of Medicine, Shahid Beheshti University of Medical Sciences, Tehran, Iran

## Abstract

Neurofibromatosis type 1 (NF1) predisposes to neural and occasionally epithelial malignancies. We report a 65-year-old man with NF1 who presented with urethral obstruction, meatal discharge, and glans swelling. Imaging identified a 22 × 25 mm hypervascular penile lesion invading the corpus spongiosum and anterior urethra. Biopsy confirmed well-differentiated squamous cell carcinoma. Staging showed no distant disease; inguinal FNA was negative. He underwent radical penectomy with perineal urethrostomy; margins were clear, and recovery was uneventful. This rare occurrence highlights RAS-pathway plausibility, supports prompt biopsy of changing genital lesions in NF1, and recommends HPV testing, molecular profiling, and multidisciplinary follow-up.

## Highlights

• Penile squamous cell carcinoma (SCC) in neurofibromatosis type 1 (NF1) is extremely uncommon, suggesting a possible link between NF1 and epithelial cancers.
• A 65-year-old man with chronic urethral stricture developed urinary obstruction, meatal discharge, and a penile mass invading the corpus spongiosum.
• Biopsy confirmed SCC; radical penectomy with perineal urethrostomy achieved clear margins and no metastasis.
• NF1-related RAS/MAPK dysregulation supports SCC risk, highlighting the need for prompt biopsy, HPV testing, and close follow-up.

## 1 Introduction

Neurofibromatosis Type 1 (NF1) is a multisystem, autosomal dominant disorder affecting approximately 1 in 4000 individuals, characterized by a loss of the neurofibromin gene, which functions as a tumor suppressor. While NF1 primarily manifests through cutaneous neurofibromas, café-au-lait spots, and optic gliomas, the condition is also associated with an increased risk for various malignancies, including malignant peripheral nerve sheath tumors, rhabdomyosarcoma, and, although rare, squamous cell carcinoma (SCC).^1^, ^2^, ^3^, ^4^

Penile squamous cell carcinoma, though it accounts for the majority of penile cancers, is less frequent in developed countries. It is strongly associated with risk factors such as human papillomavirus (HPV) infection and lack of circumcision.^4^ However, when SCC occurs in individuals with NF1, its association with other known malignancies and the role of RAS pathway hyperactivation become significant.^3^^,^^4^ While the genitourinary system, particularly the bladder, is the most commonly involved organ in NF1, direct involvement of the penis in such a context remains poorly understood.^5^ Pathological changes associated with NF1 may predispose affected individuals to develop malignancies in various regions, and this phenomenon is underscored by the reports of SCC arising in atypical sites, such as the sole of the foot, in patients with NF1.^6^

The relationship between NF1 and skin cancers, including SCC, has been explored in several studies. Individuals with NF1 have an elevated risk for developing cutaneous SCC, basal cell carcinoma, and melanoma, which is attributed to RAS signaling pathway mutations that facilitate carcinogenesis.^7^^,^^8^ Additionally, the genetic predisposition to malignancy in NF1 patients may extend beyond the skin, as evidenced by this rare case of penile SCC. The malignancy in this patient, though unusual, emphasizes the need for awareness regarding the potential for skin and other epithelial cancers in the context of NF1.

The presence of squamous cell carcinoma (SCC) in a patient with neurofibromatosis type 1 (NF1), particularly affecting the penile region, is extremely rare. This case report highlights the clinical presentation, diagnostic challenges, and treatment options for penile SCC in a 65-year-old male with NF1, contributing to the understanding of this rare but significant condition.

## 2 Case report

A 65-year-old male patient presented with a chief complaint of worsening urinary symptoms, specifically decreased force and caliber of urine, which had gradually worsened over the past years. He has a long-standing history of urethral narrowing, managed with multiple cystoscopy dilatations over the last 35 years. Approximately three weeks prior to his consultation, he developed infectious drainage from the meatus and swelling of the glans penis. The patient has also performed intermittent catheterization (CIC) for many years due to urinary retention.

He is a known case of neurofibromatosis type 1 (NF1), diagnosed based on two major criteria: freckling in the axillary regions and the presence of multiple large café au lait macules and cutaneous neurofibromas (Fig. 1-A). There is no other significant medical history, and the patient had not undergone any previous surgeries. Otherwise, the patient had been in generally good health.

**Figure 1-A:**
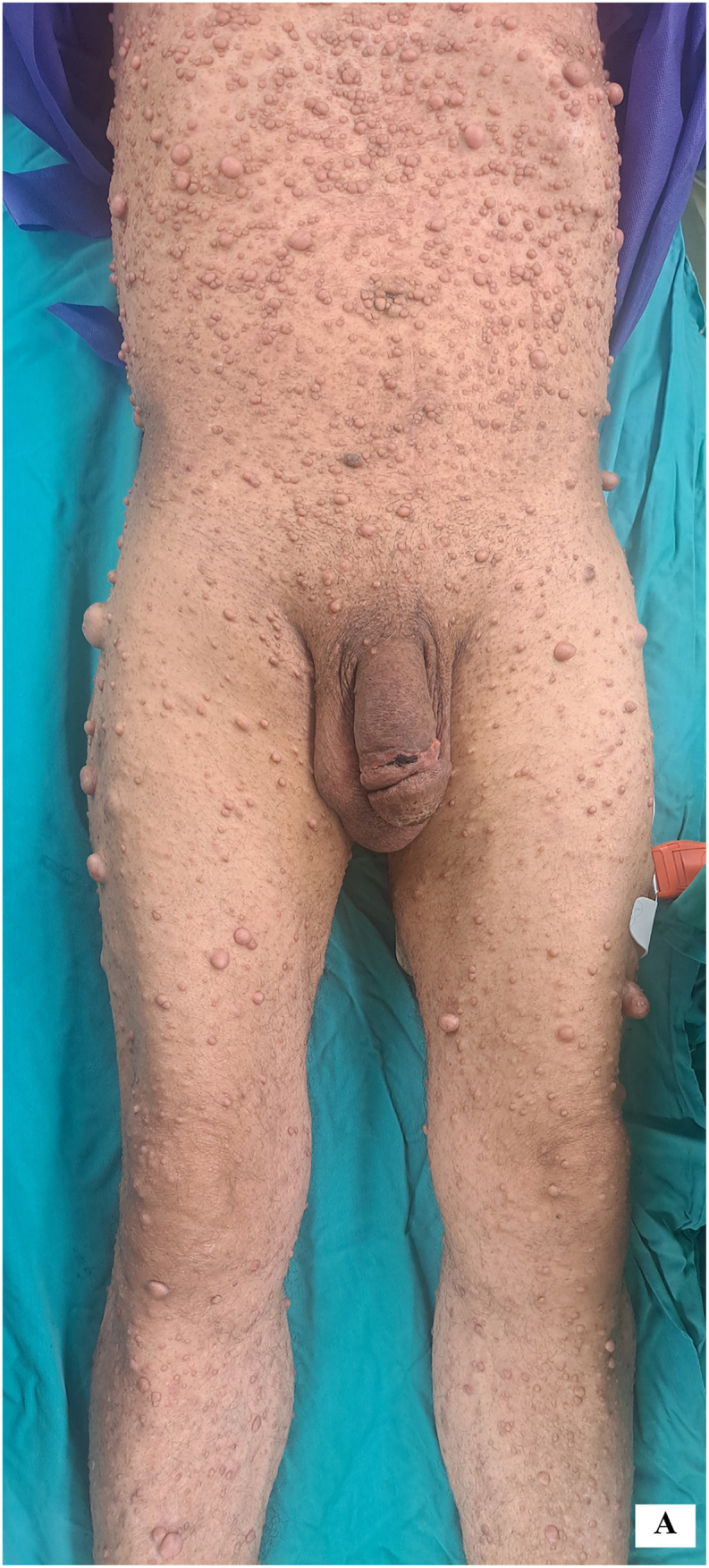
Multiple large cutaneous neurofibromas and swollen penis (pre-surgery).

Before proceeding with the surgery, the patient was consulted with an ophthalmologist, neurologist, and dermatologist to assess potential NF1-related complications. All evaluations were negative for any immediate issues that would contraindicate surgery.

Upon examination, the patient was found to have milky discharge from the meatus and a suspicious mass located just below the glans penis. There was noticeable swelling of the glans. The rest of the physical examination was unremarkable, with no other findings that warranted immediate concern.

To further evaluate the suspicious mass, a penile sonography was performed, revealing a soft tissue lesion in the anterior distal part of the penis measuring 22 × 25 mm. The lesion showed hypervascularity, which raised concerns for malignancy. A pelvic MRI was also conducted, confirming the presence of a tumor with enhancement, necrosis, and involvement of the corpus spongiosum as well as the anterior urethra, contributing to the patient's urinary retention (Fig. 1-B).

**Figure 1-B:**
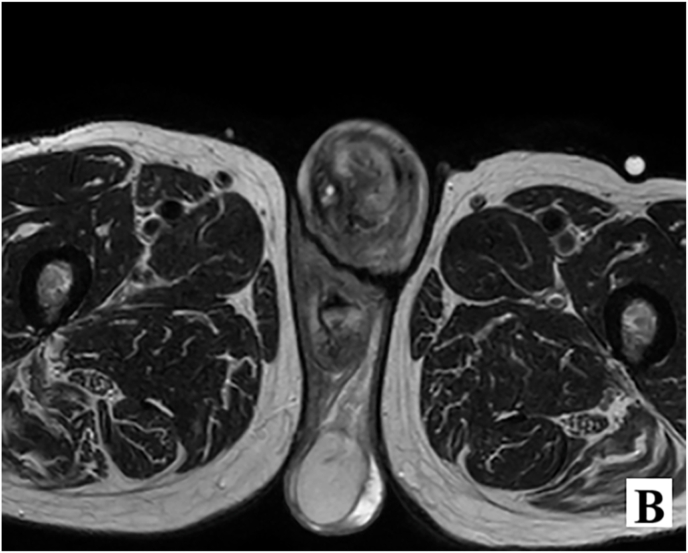
the presence of a tumor with enhancement, necrosis, and involvement of the corpus spongiosum.

Given the findings and the history of urethral narrowing and urinary retention, a cystoscopy was performed, which demonstrated significant narrowing of the whole penile urethra but no other significant abnormalities. A Foley catheter was placed to relieve urinary retention, and a biopsy was performed after degloving the penis with safe margins. Urethral discharge was also collected for cytological examination, which revealed degenerated inflammatory cells.

The biopsy pathology report confirmed the diagnosis of well-differentiated squamous cell carcinoma (SCC), Grade 1, with tumor extension into the dermis. The tumor exhibited a pushing border and a thickness of 4 mm. A skin biopsy showed inflamed skin and subcutaneous tissue, with no further signs of malignancy.

Following this diagnosis, the patient underwent a comprehensive workup, including brain and spinal MRI and IV CT scans of the chest and abdominopelvic. Fortunately, no distant metastases or positive findings were identified. Fine needle aspiration (FNA) of the inguinal lymph nodes was performed and found to be negative for tumor cells.

Given the absence of lymphatic involvement and after thorough discussions with the patient, a decision was made to proceed with radical penectomy and perineal ostomy, without lymph node dissection. During surgery, the penile specimen was initially swollen, as shown before the procedure began (Fig. 2-A). Radical penectomy was performed with an elliptical incision around the base of the penis. The fundiform and suspensory ligaments were ligated and severed. Prepubic fat was mobilized toward the specimen, and the periosteum of the pubic bone was elevated. Additionally, the periosteum of the ischiopubic rami was lifted to the inferior border to ensure adequate surgical margins (Fig. 2-B). The specimen was then excised through a lambda incision in the perineum. The deep dorsal vessels of the penis were identified, ligated, and divided. The corporeal bodies were dissected down to the tip. The dissection continued by developing a plane between the proximal urethra and corpora, and the urethra was transected, leaving a 3 cm segment for the perineal urethrostomy (Fig. 2-C). A frozen section analysis of the corpus spongiosum during the procedure confirmed clear margins, with no tumor involvement in the overlying normal skin.

**Figure 2-A:**
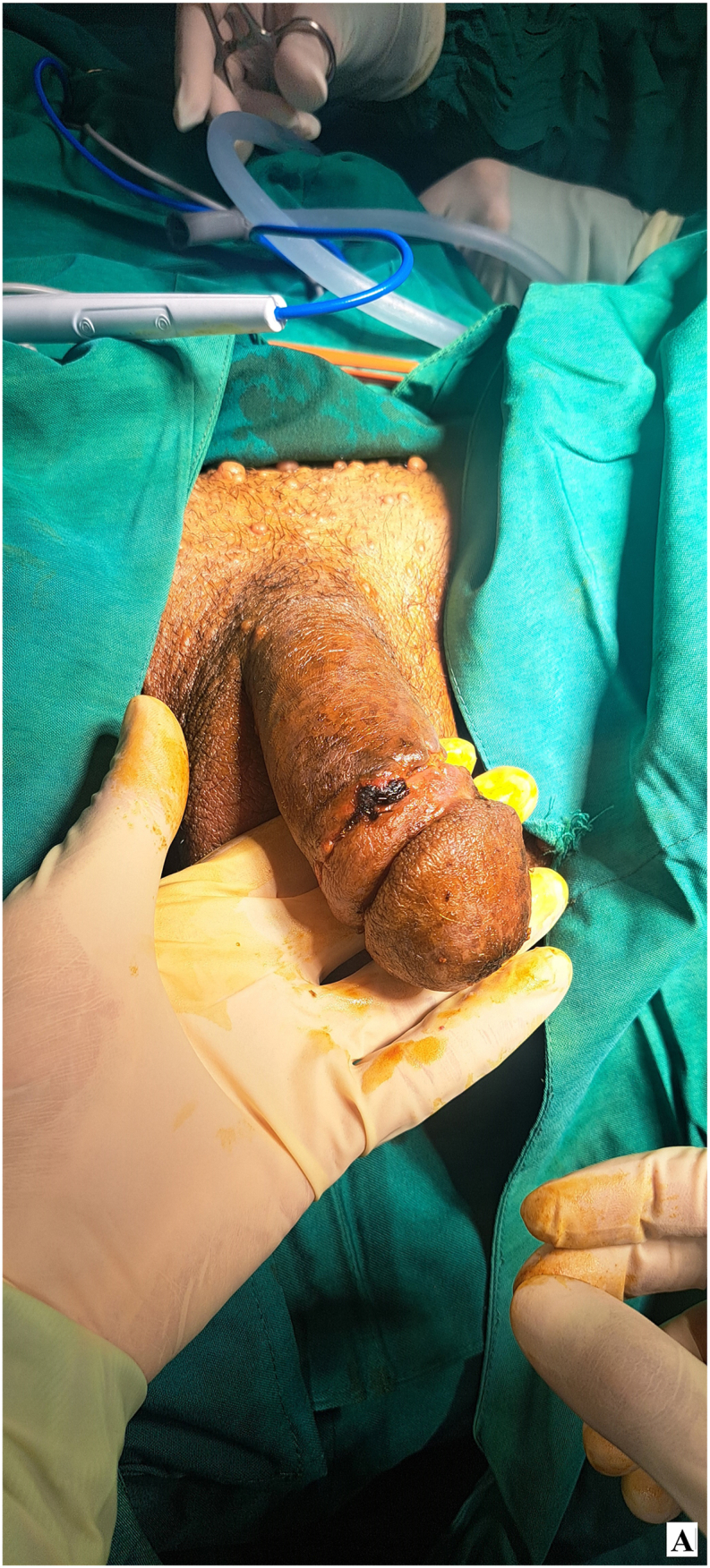
Swollen penis before starting surgery.

**Figure 2-B:**
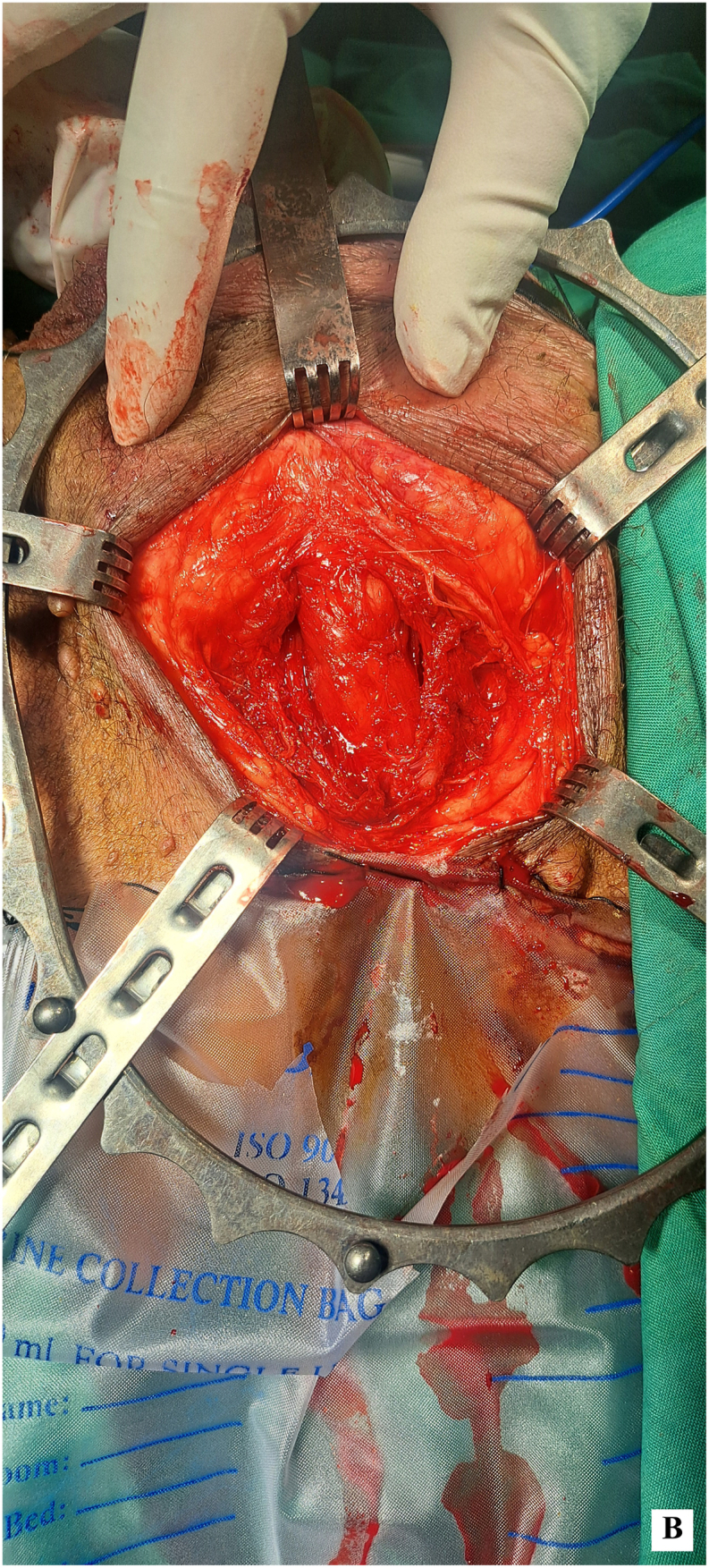
Penile degloving and urethra extraction for perineal ostomy.

**Figure 2-C:**
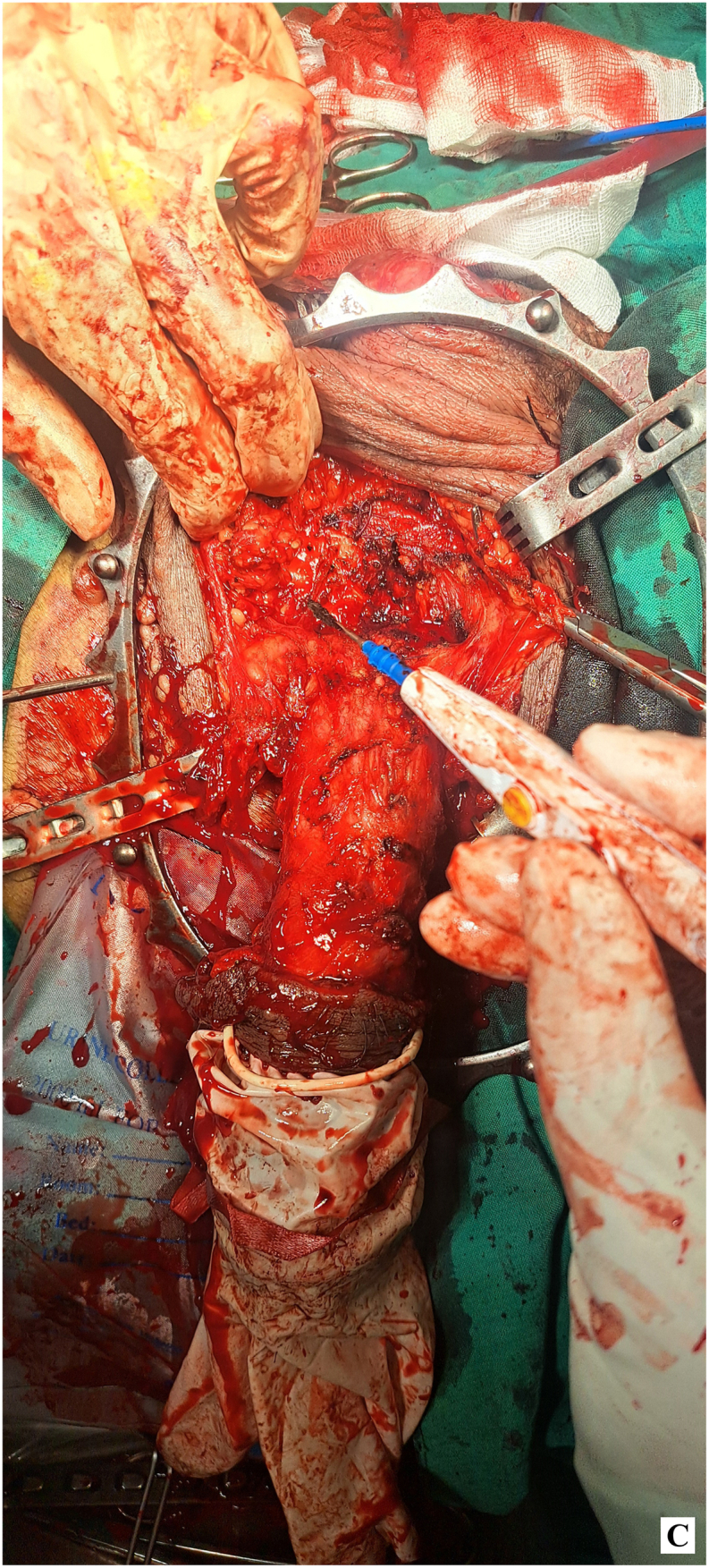
Penile resection.

After the resection, the post-surgery site, including the penectomy and perineal ostomy, was carefully documented in, taken the day after surgery (Fig. 2-D).

**Figure 2-D:**
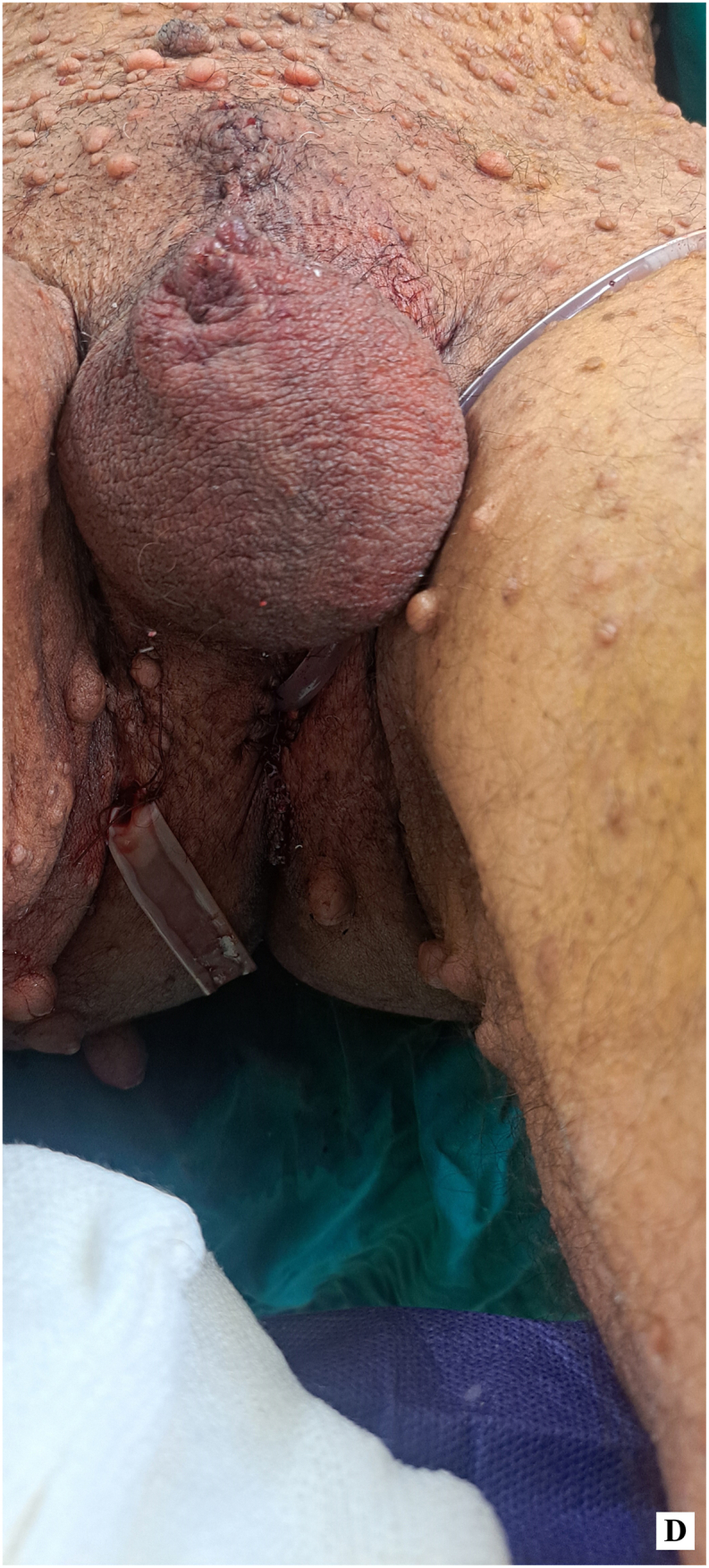
Post-surgery penectomy site and perineal ostomy (taken the day after surgery).

The patient's post-operative recovery has been uneventful, with no immediate complications. Regular follow-up visits are planned to monitor for any recurrence of the tumor or development of new symptoms. Given the clear surgical margins and absence of lymph node involvement, the prognosis is favorable at this stage.

The final pathology confirmed the presence of moderately differentiated SCC with invasion of the corpus spongiosum, classified as pathological stage PT3NxMx. The surgical margins were tumor-free, though the overlying penile skin was involved.

The gross examination of the specimen revealed tumor involvement in the distal section (Fig. 3-A), while the proximal portion of the specimen showed no tumor involvement (Fig. 3-B).

**Figure 3-A:**
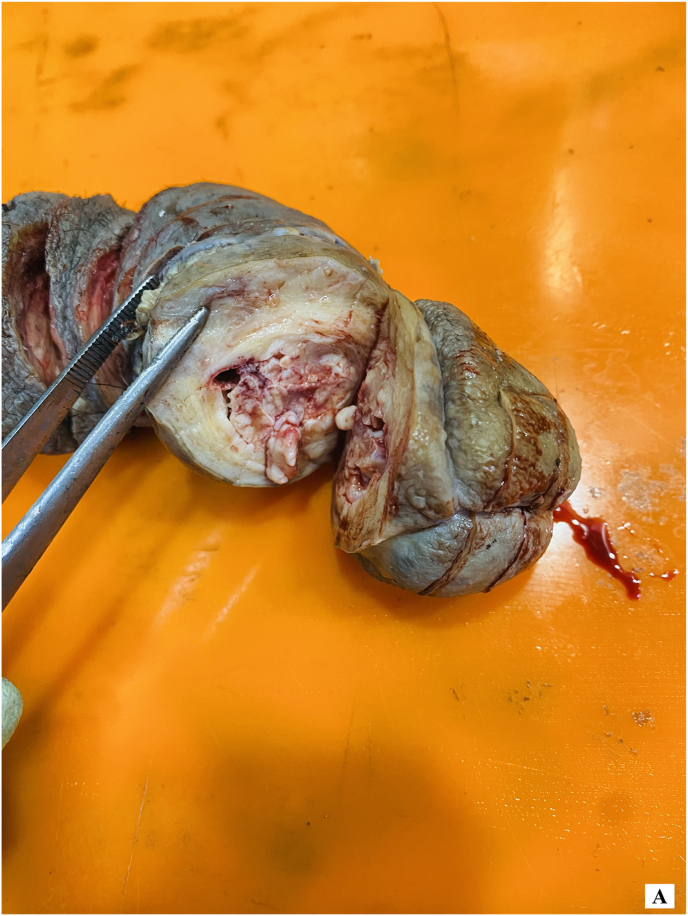
Gross examination of specimen showing involvement of tumor.

**Figure 3-B:**
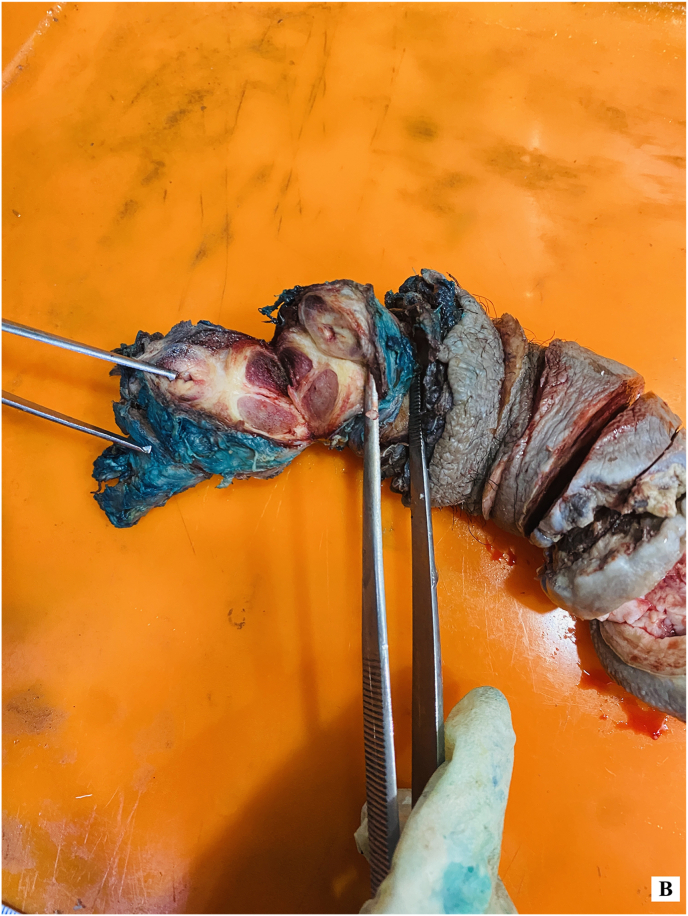
Gross examination of specimen showing no involvement.

Further pathological examination revealed islands of squamous epithelial cells invading the corpus spongiosum (Figure 4-A, Figure 4-B-A and Fig. 4-B).

**Figure 4-A:**
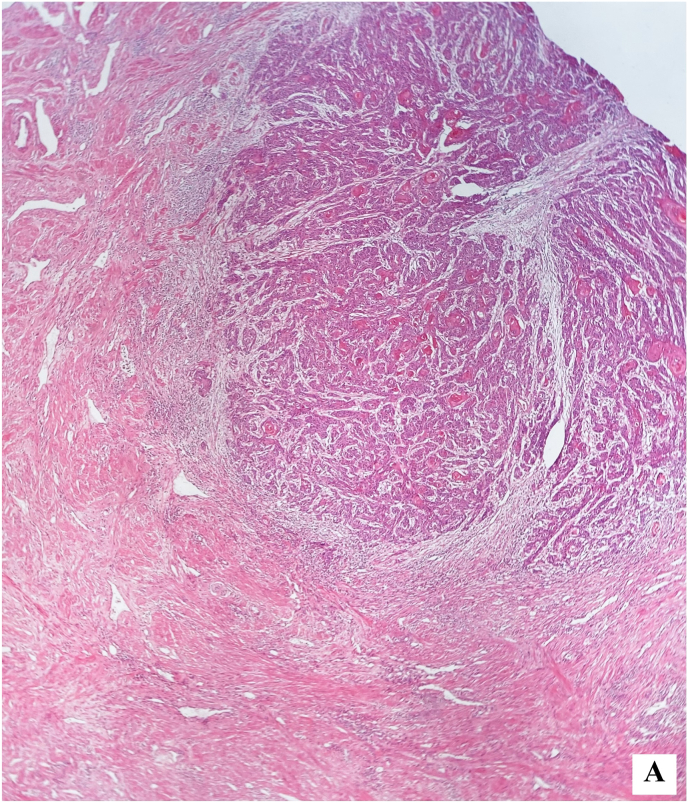
Islands of squamous epithelial cells invading the corpus spongiosum.

**Figure 4-B:**
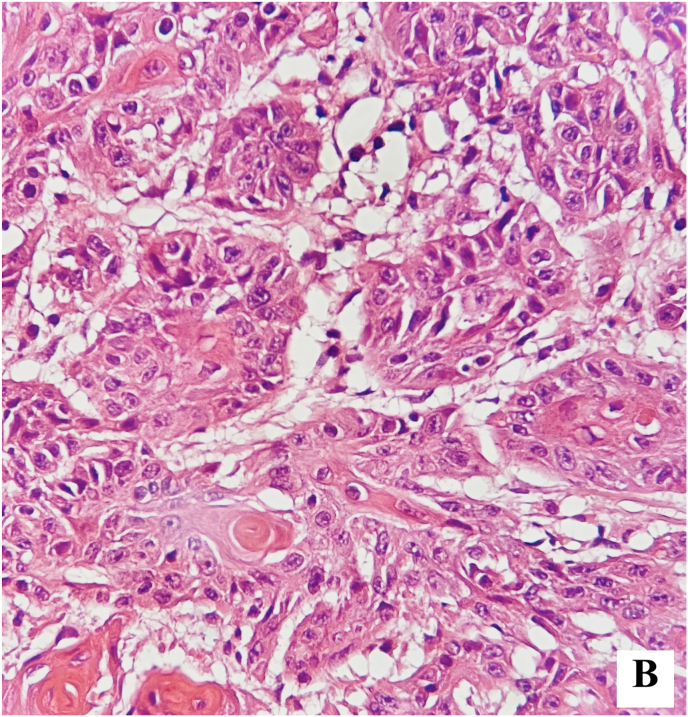
Islands of squamous epithelial cells.

Given the absence of lymphatic involvement and negative surgical margins on both the penile skin and corpus spongiosum, the patient's prognosis is favorable. Post-operative follow-up will include regular clinical examinations every 3–6 months for the first 2–3 years to monitor for signs of recurrence, along with imaging (chest CT and abdominal/pelvic scans) as clinically indicated. As there is no indication of metastasis, adjuvant radiation, chemotherapy, or immunotherapy are not currently required. Long-term follow-up will focus on monitoring the perineal urethrostomy, ensuring proper care and preventing complications, while also addressing the patient's psychological and sexual health. With no recurrence over time, the follow-up visits will gradually decrease in frequency, moving to every 6–12 months after 3 years, and the risk of recurrence after 5 years will be significantly reduced. Overall, the patient's outlook remains positive, with close monitoring ensuring any potential recurrence is detected early.

## 3 Discussion

This report describes a rare occurrence of locally advanced penile squamous cell carcinoma (SCC) in a patient with neurofibromatosis type 1 (NF1). Although NF1 classically predisposes to neurogenic tumors, large NF1 cohorts and registry studies demonstrate that patients with NF1 develop a broader spectrum of neoplasms often at younger ages and, for some histologies, with worse disease-specific survival highlighting the clinical importance of non-neurofibroma malignancies in this population.^9^^,^^10^

The biological plausibility linking NF1 to epithelial cancers rests on neurofibromin's role as a negative regulator of RAS signaling. Germline NF1 loss and somatic NF1 alterations result in RAS/MAPK pathway hyperactivation, a central driver of cell proliferation and genomic instability that is implicated across multiple tumour types. Next-generation sequencing studies show somatic NF1 aberrations in diverse sporadic malignancies, supporting a mechanistic rationale whereby NF1 deficiency can contribute to epithelial carcinogenesis and to therapeutic resistance in certain contexts.^11^, ^12^, ^13^

Penile involvement in NF1 is uncommon. Within the genitourinary tract, bladder lesions are the most frequently reported NF1 manifestations, whereas penile neurofibromas and penile malignancies have only been described sporadically in the literature. This rarity can delay recognition of an epithelial malignancy when a penile mass arises in an NF1 patient; thus, changes in size, new ulceration, discharge, or obstructive urinary symptoms should prompt expedited imaging and biopsy to distinguish neurogenic lesions from primary penile cancer.^1^^,^^14^, ^15^, ^16^

Management in our case followed oncologic principles appropriate for locally advanced disease. Tumour extension into the corpus spongiosum and anterior urethra places lesions at T3 or higher, for which partial or total penectomy with perineal urethrostomy is an accepted option when organ preservation cannot secure negative margins. Careful nodal assessment is critical because inguinal node status is the dominant prognostic factor in penile cancer; for clinically node-negative but intermediate- or high-risk primaries, dynamic sentinel node biopsy or modified inguinal lymphadenectomy are guideline-recommended approaches. Close postoperative surveillance during the first 2 years is warranted given the timing of most recurrences.^17^, ^18^, ^19^

Two practical points are important for clinicians and future reports. First, because human papillomavirus (HPV) drives a subset of penile SCCs and carries prognostic implications, HPV (and p16) testing should be obtained when tissue is available. Second, whenever feasible, molecular profiling of the tumour (including assessment for somatic NF1 and other RAS-pathway alterations) can provide etiologic insight and may, in future, help identify targeted therapeutic opportunities for unresectable or metastatic disease. Although specific NF1-targeted systemic therapies for epithelial tumors are not yet established, pathway-directed agents (for example, MEK inhibitors) are an area of active investigation in NF1-related neoplasia.^11^^,^^13^^,^^17^

Limitations of this case include the absence of tumor HPV/p16 testing and somatic NF1 profiling; these data would strengthen any mechanistic link between NF1 and penile SCC. Nevertheless, the case reinforces an important clinical message: clinicians caring for patients with NF1 should maintain a low threshold for biopsy of new or changing mucocutaneous and genitourinary lesions.

## 4 Conclusion

Penile SCC arising in an NF1 patient is rare but biologically plausible given RAS/MAPK dysregulation in NF1. Prompt biopsy, guideline-based surgical management with careful nodal assessment, HPV testing when possible, and consideration of molecular tumor profiling are recommended. Aggregation of additional cases and inclusion of molecular data will be needed to clarify whether NF1 confers a reproducible, site-specific excess risk for epithelial cancers such as penile SCC.

## CRediT authorship contribution statement

**Babak Javanmard:** Writing – original draft, Validation, Supervision, Project administration, Investigation, Conceptualization. **Sina Samenezhad:** Writing – review & editing, Writing – original draft, Visualization, Validation, Supervision, Project administration, Methodology, Investigation, Conceptualization. **Dorna Rafighi:** Validation, Resources, Formal analysis, Data curation. **Lena yaghoubpour:** Visualization, Investigation, Data curation. **nasim Dehghan:** Visualization, Resources.

## Patient consent

Patient informed consent was obtained to publish his information. The patient's private information remained confidential with the researchers.

## Ethical approval

This study was reviewed by our hospital Institutional Review Board (IRB) and was deemed exempt from formal ethical approval because the treatment and data collection were based entirely on established clinical guidelines and standard urological practice performed by an expert urologist. No experimental interventions or novel protocols were involved, and all actions followed evidence-based, routine medical care.

## Funding

This research received no specific grant from any funding agency in the public, commercial, or not-for-profit sectors.

## Declaration of competing interest

The authors declare no competing financial or personal interests.
